# Multimodal sensory processing in *Caenorhabditis elegans*

**DOI:** 10.1098/rsob.180049

**Published:** 2018-06-20

**Authors:** Athanasios Metaxakis, Dionysia Petratou, Nektarios Tavernarakis

**Affiliations:** 1 Institute of Molecular Biology and Biotechnology, Foundation for Research and Technology Hellas, Nikolaou Plastira 100, Heraklion 70013, Crete, Greece; 2 Department of Basic Sciences, Faculty of Medicine, University of Crete, Heraklion 71110, Crete, Greece

**Keywords:** *Caenorhabditis elegans*, behavioural plasticity, multisensory processing, interneuron, sensory integration

## Abstract

Multisensory integration is a mechanism that allows organisms to simultaneously sense and understand external stimuli from different modalities. These distinct signals are transduced into neuronal signals that converge into decision-making neuronal entities. Such decision-making centres receive information through neuromodulators regarding the organism's physiological state and accordingly trigger behavioural responses. Despite the importance of multisensory integration for efficient functioning of the nervous system, and also the implication of dysfunctional multisensory integration in the aetiology of neuropsychiatric disease, little is known about the relative molecular mechanisms. *Caenorhabditis elegans* is an appropriate model system to study such mechanisms and elucidate the molecular ways through which organisms understand external environments in an accurate and coherent fashion.

## 1. Introduction

Organisms must sense and ‘understand’ external stimuli in order to adapt to continuously changing natural conditions. Adaptability is largely dependent on the ability of the nervous system to receive and integrate information regarding physical parameters, such as temperature and humidity, food availability, presence of predators and sex pheromones, so that it can orchestrate proper physiological and behavioural responses to ensure survival and reproduction. Diversity of physical and biological factors that affect organisms has led to the evolution of several neuronal circuits that accomplish perception of various sensory modalities, such as temperature, vision, taste, smell, touch and hearing. Sensory neurons receive external information that is processed and integrated to regulate behaviour and form memories. Each environmental stimulus can trigger multiple sensory neurons and generate various sensory cues, which must be integrated and assessed by the nervous system. Nevertheless, the stimuli that an organism must perceive and process in order to better confront natural challenges can be highly complex, and simultaneous perception of different stimuli is necessary for the construction of a comprehensible depiction of habitats and a fully featured understanding of natural conditions.

Often organisms must choose between opposing sensory signals in nature. An organism with enhanced food-searching activity or copulating behaviour is under an increased risk to become prey of its predators or face adverse physical microenvironments that can kill it. To make the best decision for its survival and efficient reproduction, an organism must receive as much information as possible regarding the relative degree of danger through its sensory neurons. Subsequently, this heterogeneous information must be integrated and processed into decision-making neuronal centres to regulate relative responses [1]. Such decision-making centres must consider the organism's physiological status, e.g. the level of hunger or food shortage, to judge if the enhanced risk for survival is necessary and accordingly regulate the behavioural response [2,3]. This presupposes the capacity of decision-making centres to sense organism's physiological state and initiate behavioural responses through modulation of executive neurons. Hence, decision-making neurons can serve not only as sensors of external and internal stimuli, but also as behavioural modifiers.

Several studies suggest the existence of decision-making centres that accomplish responses to multisensory cues in all animals tested so far. In *Drosophila melanogaster*, visual and chemosensory inputs converge into the mushroom bodies to potentiate plasticity in courtship [4]. In primates, cerebral cortex integrates and assesses information from sensory inputs to modulate behavioural responses [5]. The above and several more studies suggest the existence of defined neuronal domains that integrate multisensory information and serve as decision-making centres. Whether multisensory convergence occurs within particular brain regions (areal convergence) or within specific neurons (neuronal convergence) is unknown [1]. Instead, other studies suggest the existence of multiple multisensory integration centres in higher organisms [6,7]. To date, the enormous complexity of the nervous system in higher animals makes functional mapping of the brain impossible and the elucidation of mechanisms governing multisensory processing a difficult task.

Recent research on multisensory integration has focused on *Caenorhabditis elegans*, a well-studied nematode with a simple nervous system, comprising only 302 neurons. With 6393 chemical synapses, 890 gap junctions and 1410 neuromuscular junctions detected and its synaptic wiring fully reconstructed [8–11], research on *C. elegans* enables the functional and molecular characterization of single neurons. Moreover, a large arsenal of molecular tools facilitates genetic and behavioural manipulations and analysis. Furthermore, novel techniques, such as calcium imaging, can directly link activation of individual neurons to specific sensory stimuli [12–15]. Hence, *C. elegans* is a proper animal model to dissect mechanisms regulating multisensory integration in complex organisms such as humans.

## 2. Multisensory perception in *Caenorhabditis elegans*

### 2.1. Sensory neurons in *Caenorhabditis elegans*

*Caenorhabditis elegans* has a simple sensory system, consisting of 60 ciliated sensory neurons that sense chemical, olfactory, thermal and mechanical stimuli and relative position of the body (proprioception). Three groups of sensory neurons participate in the identification of chemical cues, the amphids and the inner labial neurons in the head and the phasmids in the tail [16,17]. The neurons with the most prominent role in identifying gustatory stimuli are the ASE. ASE neurons together with ASH mainly, and to a lesser extent ASI, ADF, ASG, ASJ, ASK, ADL and IL2 in the head and PHA and PHB in the tail, recognize water soluble attractants and repellents [18]. Chemotaxis to volatile odorants is mediated by the olfactory neurons AWA, AWB and AWC [19] and the polymodal neuron ASH [20]. AFD, BAG and ASE neurons sense CO_2_, while AQR, PQR and URX neurons are mainly O_2_ sensors and weak CO_2_ sensors [21]. The circuit that senses oxygen also includes SDQ, ALN, PLN, ADL and ASH neurons [22,23]. The main sensory neurons that respond to temperature changes are the AFD neurons, though AWC, ASI, FLP and PHC also participate in thermosensation [24,25]. Low noxious temperatures are perceived by PVD neurons [26]. ADL, ASH and AWB neurons respond to several repulsive stimuli to produce avoidance behaviour [27,28]. These stimuli include hyperosmolarity, mechanical stimuli and volatile repellents. By contrast, sensory neurons called AWA, AWC and ASE are involved in responses to an attractant [19,28]. Moreover, ASH together with ASJ, AWB and ASK neurons mediate light avoidance and electrosensory navigation [29,30]. Thirty sensory neurons have been identified in hermaphrodites to respond to mechanical stimuli. These are the ALM, PLM, AVM, PVM, PVD, ADE and PDE touch receptor neurons found at the midbody of *C. elegans* and the ASH, FLP, OLQ, CEP and IL1 neurons found at the nose tip [26,31–33].

### 2.2. Sensory transduction

The above sensory receptors are specialized for certain modalities, which are converted to neuronal signals. In *C. elegans*, the mechanisms facilitating sensory transduction of single stimuli have been studied through genetic and behavioural studies [34,35]. Binding of a chemical ligand or external force on receptor proteins located at the surface of sensory cells provokes conformational changes that, depending on their relative strength, can lead to the induction of intracellular chemical alterations. Such alterations can subsequently lead to the generation of electrical signals, through which sensory information is transferred to the nervous system. Sensory receptor families with chemosensory and mechanosensory functions are the degenerin/epithelial Na^+^ channel (Deg/ENaC) family, the transmembrane channel-like proteins and ionotropic receptors [18,36–41]. Several sensory receptors are well characterized, such as the odorants-specific G protein-coupled receptors [42,43], the mechanosensory TRP receptors of the NOMPC family [40] and the Deg/ENaC ion channel receptors that are activated by mechanical stimuli [36–38,41,44,45].

### 2.3. Polymodality of sensory neurons

In *C. elegans*, avoidance responses require either unimodal or polymodal sensory neurons. In the latter case, single sensory neurons are able to perceive stimuli from various modalities. Such neurons are the nociceptors, sensory neurons that detect intense and putatively harmful mechanic, thermal or chemical stimuli [46]. A well-studied example of avoidance response in *C. elegans* involves the pair of ASH neurons. They are located at the nose and they are responsible for sensing and conducting avoidance responses against high osmotic strength, low pH, food odours, nose touch, heavy metals and alkaloids [27,33,47]. A reasonable question arising is how ASH neurons coordinate aversive responses to different stimuli. Studies in the previous decade have shown that ASH neurons activate different synaptic pathways to regulate responses against mechanical and osmotic stimuli [35,48,49]. Combined genetic, electrophysiological and behavioural analyses showed that this is achieved through differential activation of postsynaptic NMDA and non-NMDA receptors. Specifically, although mechanical stimulation activates only synaptic non-NMDA receptors, osmotic stimuli induce a much higher secretion of synaptic glutamate that is capable of activating not only non-NMDA but also extrasynaptic NMDA receptors. As a result, the same sensory neurons can sense distinct modalities and adjust behavioural responses through different synaptic outputs. Interestingly, polymodality of sensory neurons also characterizes other organisms. In *Drosophila*, antennal nerves respond to ammonia, but also to air humidity [50,51]. In mice, olfactory sensory neurons respond to both odours and pressure changes [52]. Hence, polymodality of sensory neurons is a conserved mechanism through which single neurons broaden their sensory capacity and facilitate multisensory integration.

### 2.4. Co-action of sensory neurons

Sensory neurons can also collaborate to sense external stimuli. A well-studied paradigm is the sense of carbon dioxide [21,53–55]. The main sensory neurons for sensing CO_2_ are the AFD and BAG neurons. However, their activity is not sufficient to induce a repulsive behaviour. Degree of repulsion is dependent, among others, on ambient oxygen-sensing neurons, the URX neurons. Worms with a mutation reducing expression of the neuropeptide receptor NPR-1 are insensitive to CO_2_. Carefully designed experiments have shown that NPR-1 receptor inhibits oxygen-sensing URX neurons, which are also activated by increases in ambient oxygen [23,56]. Ablation of the URX neurons in *npr-1* mutants restores CO_2_ avoidance, suggesting that NPR-1 enables CO_2_ avoidance by inhibiting URX neurons. Moreover, in *npr-1* mutants, oxygen-induced activation of URX inhibits CO_2_ avoidance. Hence, CO_2_ avoidance requires either low O_2_ presence or inactivity of URX neurons.

In another example, worms respond to moisture gradient through the combinatorial action of both mechano- and thermosensory neurons. Specifically, the mechanosensory FLP neurons sense the level of hydration-mediated subcuticular stretching via the DEG/ENaC/ASIC mechanoreceptor complex. This information is combined with thermal cues caused by humidity-mediated evaporative cooling that is generated by stimulation of cGMP-gated channels in the thermosensory AFD neuron pair [57]. Thus, hygrosensation in *C. elegans* requires the integration of both mechanical and thermal cues.

### 2.5. Crosstalk of sensory neurons

Sensory neurons are also able to cross-modulate their activity. *Caenorhabditis elegans* senses odours intensity through the combinatorial activity of primary and secondary neurons that crosstalk through neuropeptides signalling. For example, although ASE sensory neurons are responsible for salt detection, dramatic changes in salt concentration are sensed through recruitment of AWC olfactory neurons. This is achieved through the release of INS-6 insulin-like peptide by activated ASE neurons, which, in turn, modulates AWC neurons [58]. Hence, the combined action of ASE and AWC neurons adjusts sensing of high salinity and relative responses. In another example, AWC and AWA neurons sense the food odour benzaldehyde and secrete insulin-like peptides and acetylcholine, to target and sensitize ASEL and AWB neurons [59]. Concerted action of the above neurons is necessary for attraction to benzaldehyde. In conclusion, sensory neurons have the capacity to decode multisensory stimuli through polymodality, simultaneous activity or cross-modulation, and through these mechanisms sensory neurons increase their capacity to fine-tune multisensory integration and provoke relative behavioural responses.

## 3. Interneurons: the decision-making centres in *Caenorhabditis elegans*

**Figure 1.**
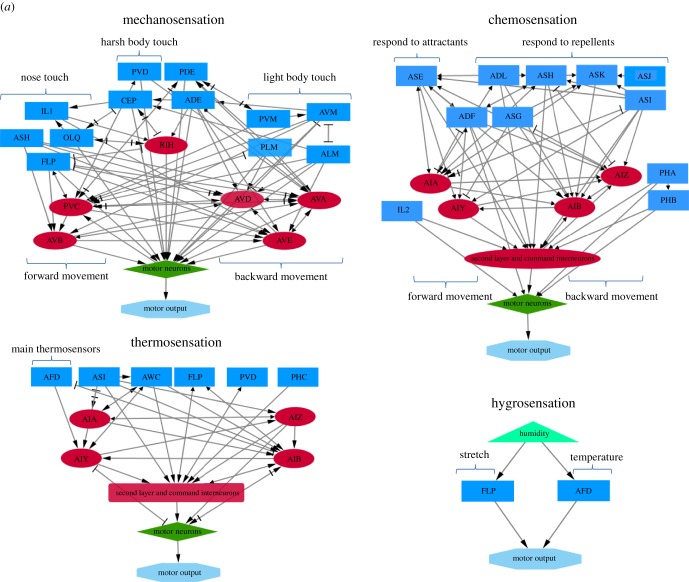

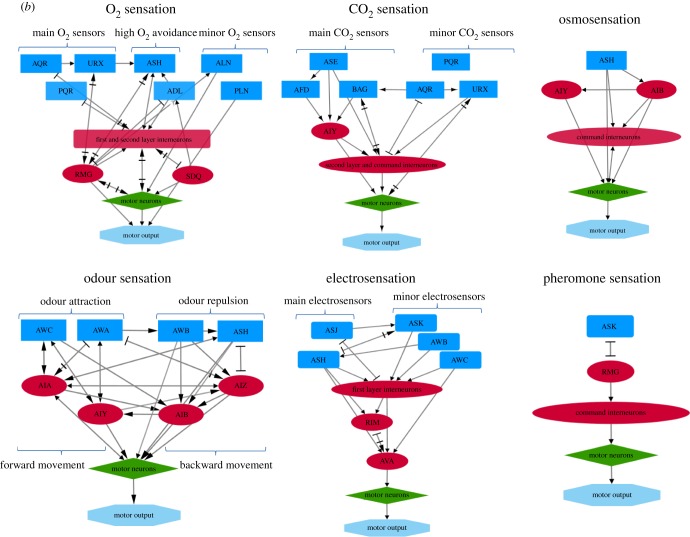
Neural circuits of *C. elegans* sensory processing. Sensory neurons are indicated with blue rectangles, interneurons with red ellipses, motor neurons with green diamonds and motor output with light blue octagons. Light green triangles indicate sensory stimuli. First layer interneurons are characterized as those that are postsynaptic to sensory neurons, second layer as those that are presynaptic to command interneurons and command interneurons as those that are presynaptic to motor neurons. (*a*) Neuronal wiring diagrams for mechanosensation, chemosensation, thermosensation and hygrosensation. (*b*) Neuronal wiring diagrams for O_2_ and CO_2_ sensation, osmosensation, electrosensation, pheromone sensation and odour sensation. Arrows denote chemical synapses, while bars denote electrical synapses (gap junctions). Strength and type (excitatory or inhibitory) of the synapse are not indicated. Interactions can be retrieved from http://wormweb.org/neuralnet [60].

Organisms need to combine information from various sensory modalities to achieve a more coherent and composite understanding of natural environments. This complex flow of information, derived from multiple stimuli, must be integrated into centralized neurons, to be processed and trigger relative behavioural responses. Anatomical but also genetic and behavioural data suggest that information from sensory neurons is transferred and processed into a distinct category of nerve cells, the interneurons (figure 1). A set of five interneurons has been shown to integrate responses to mechanical stimuli and affect the locomotor behaviour, AVB, PVC, AVA, AVD and AVE [8,31,61]. Concerning chemotaxis, activity of AIY interneurons alone is sufficient to mediate chemotactic responses, mainly by promoting forward movement and gradual turnings [62]. However, AIA, AIB and AIZ neurons also participate in the formation of attraction or avoidance behaviours to water soluble attractants [63–65]. Apart from the integration of gustatory stimuli, AIY together with AIZ, AIB, AIA and RIA mediate responses to thermal stimuli [66], while AIY and AIB mediate responses to olfactory stimuli and osmotic changes [67]. AIY and RIA interneurons participate in the regulation of avoidance or attraction by CO_2_ [68], while RMG interneurons participate in oxygen sensation [69]. RIM and AVA interneurons are implicated in electrosensory detection [30]. In conclusion, several studies support that interneurons are the convergence sites of multisensory inputs from sensory neurons and that they serve as coincidence detectors [70].

A well-studied example in *C. elegans* is the AIA interneuron, which is the decision centre of behavioural choice between the attractive odorant, diacetyl, and an aversive stimulus, Cu^2+^ ions. Diacetyl is sensed by the AWA sensory neurons and Cu^2+^ ions are sensed by the polymodal sensory neurons ASH. The AIA interneuron is postsynaptic of ASH and connected with AWA through gap junctions. Combined genetic and behavioural analyses revealed that integration of the two opposing sensory cues is dependent on AIA neurons and, specifically, on the conflicting pathways GCY-28/CNG-1 and HEN-1/SCD-2, which function in AIA interneurons and modulate their activity [64,71]. According to the proposed model, the AIA interneurons regulate activity of the AIB interneurons through inhibitory synapses. The latter induce avoidance behaviours [67]. Hence, the AIA neurons are likely to promote attraction to odours through inhibition of the AIB neurons. Other studies also indicate a role for the GCY-28/CNG-1 and HEN-1/SCD-2 pathways in multisensory integration of opposing sensory cues [72]. As in the case of salt chemotaxis learning, the GCY-28/CNG-1 and HEN-1/SCD-2 pathways are also shown to modulate food-associated thermotactic behavioural plasticity [64,73,74].

In another example, octanol, an aversive odorant, is sensed by ASH neurons which initially activate AIB interneurons through glutamatergic synapses to promote avoidance behaviour. However, in the presence of food, octanol does not repel worms. AIB interneurons receive synaptic signals from both the ASH and AWC sensory neurons. The food odour-sensing AWC and salt-sensing ASER neurons can activate and deactivate, respectively, AIB through distinct glutamatergic transmissions. Upon the presence of food, worms finally move towards octanol. Food inhibits AWC neurons and their positive effect on AIB activity. Moreover, ASER neurons deactivate AIB. Hence, although octanol initially activates AIB interneurons and avoidance responses, food odours and salt inhibit AIB activation and, consequently, abrogate octanol-evoked avoidance behaviour [75].

### 3.1. The hub and spoke circuit

Animals need to respond acutely and accurately to environmental threats and stimuli. Research in *C. elegans* has revealed a mechanism through which worms respond acutely to multisensory inputs that regulate social behaviour in worms. Specified neuronal circuits underlie social behaviour and facilitate rapid responses to environmental stimuli that affect aggregation and other aspects of social behaviour [76]. In such a circuit, the ASK sensory neurons, among others, sense pheromones and connect to a single pair of interneurons, the RMG neurons. Sensory neurons are also interconnected through electrical synapses and this complex circuit can strengthen coincidence responses through lateral facilitation. Pheromones-sensing neurons and RMG interneurons are connected with gap junctions, thus allowing their direct metabolic and electrical communication. High RMG activity enhances ASK responses in social strains, causing hermaphrodite attraction to pheromones at concentrations that repel solitary hermaphrodites. Also, solitary strains differ from social strains in the activity of the neuropeptides receptor gene *npr-1* which mainly acts at the RGM interneurons. Hence, social attraction in *C. elegans* is mainly regulated by a neuronal circuit that largely resembles a ‘hub and spoke’ circuit, in which RMG interneurons have the role of the ‘hub’ and sensory neurons have the role of the ‘spoke’. Such a system facilitates the integration of multiple sensory cues and the rapid response of worms to population density and the presence of mates [70,76].

Another example of a ‘hub and spoke’ circuit has been described to regulate the nose touch response [33]. Here, three sensory neurons, ASH, FLP and OLQ, sense touch to the nose and activate RIH interneurons through gap junctions. In this case, the three sensory neurons serve as ‘spoke’ neurons and the RIH interneurons serve as the ‘hub’ of the circuit. Sensory neurons interact with each other and this interaction modifies the electric stimulus that gets transferred to the ‘hub’ neuron [77,78]. Hence, the formation of gap junctions between sensory neurons and interneurons and the anatomical pattern of ‘hub’ and ‘spoke’ circuits are common mechanisms for the facilitation of multisensory integration and the relative behavioural response.

## 4. Biogenic amines and neuropeptides modulate responses to multisensory inputs

Organisms take decisions depending on their internal physiological state. Hunger, stress and health condition are some of the factors that modulate their responses to external stimuli. Internal physiological state affects expression and release of neuromodulators, molecules that can act from a distance on nerve cells and can have a general effect on neuronal circuits. In a previously described multisensory integration circuit, behavioural response to octanol is mediated through activity of AIB interneurons [75]. Food and serotonin modulate this circuit through different modes. Smell of food and serotonin, which is increased upon feeding, deactivate AIB and avoidance behaviour. Several examples show that, except for serotonin, other biogenic amines also regulate neuronal circuits that underlie multisensory integration and relative behavioural responses [70]. Dopamine serves as a signalling molecule that affects avoidance and food-searching behaviours [79,80]. Tyramine, another biogenic amine that represents internal metabolic state of *C. elegans*, regulates threat tolerance [81]. When worms must cross a hyperosmotic barrier to reach food sources, the choice is made by the RIM interneuron. RIM innervates ASH sensory neurons with tyraminergic inputs. High levels of tyramine represent a well-fed state for worms. When tyramine levels are adequate, ASH neurons are activated and promote avoidance behaviour and backwards movement. Under low tyramine levels, ASH neurons are inactivated and, as a result, osmosensitivity is decreased. This causes the worms to move towards the food source, without being constrained by the hyperosmotic barrier. Biogenic amines levels indicate internal metabolic state in animals and the modulation they exert on multisensory integration is crucial for homeostasis maintenance.

Interestingly, circuits involving different biogenic amines seem to interact to control feeding behaviour. Serotonergic NSM neurons promote feeding in the presence of attractive odours, though tyraminergic RIM interneurons inhibit feeding in the presence of aversive cues. These circuits are shown to interact with each other and the outcome of this interaction determines feeding behaviour [82].

Except for biogenic amines, neuropeptides are also shown to affect multisensory integration and behavioural output. Neuropeptides act as neuromodulators and they can facilitate interaction between distant interneurons and/or sensory neurons. There are several examples showing a regulatory role for neuropeptides on activity of interneurons. AIA interneuron is regulated by HEN-1, which is produced by another interneuron, AIY [64,71]. Chalasani *et al.* [83] identified a neuropeptide-to-neuropeptide feedback loop that controls sensing ability in primary olfactory neurons. In AWC olfactory neurons, expression of NLP-1 neuropeptide reduces AWC activity. NLP-1 binds the NPR-11 receptor, which is located at the postsynaptic AIA interneurons. The latter, in turn, releases INS-1 neuropeptide that modulates sensitivity to odours in AWC neurons [83]. In another study, insulin and NPR-1 neuropeptides were found to regulate and fine-tune chemosensation through affecting the expression of receptor genes in chemosensory neurons [84]. Hence, neuropeptides play a major regulatory role on multisensory integration through affecting activity of sensory neurons and interneurons, and also through facilitating interaction among interneurons.

## 5. Deficient multisensory integration and human diseases

Functional multisensory integration has a strong impact on the ability of organisms to understand their complex environment and to sufficiently react against external stimuli. Several findings support that inability to properly integrate environmental cues might lead to neuropsychiatric disorders in humans, such as autism, schizophrenia and attention deficit hyperactivity disorder (ADHD) [85–87]. Interestingly, these disorders are characterized by deficient sensory processing and by common comorbidity [88–92]. Although relative mechanisms are still unknown, several lines of evidence suggest a link between certain neuropsychiatric disorders and dysfunctional sensory integration.

Autism spectrum disorders (ASDs) are associated with altered multisensory processing and inability to integrate multisensory inputs into a unified percept [93–95]. In mouse models of ASD, multisensory integration is impaired. This is possibly due to impaired integration in the insular cortex, a brain centre where sensory, emotional and cognitive information is converged [96–99]. In support, recent evidence suggests specific neuronal pathways underlying multisensory dysfunction in children with ASD [100,101]. Specifically, a gain-of-function coding variant in the serotonin transporter (SERT) is associated with sensory aversion in humans. Upon its expression in mice, it induces phenotypes reminiscent of ASD, such as deficient social and communicative function and repetitive behaviours. Furthermore, these mice exhibit behavioural deficits in multisensory function that extend beyond changes in unisensory performance [102]. Hence, strong indications suggest that dysfunctional multisensory integration underlies, at least in part, ASDs.

Recent studies show that schizophrenic patients exhibit altered integration of distinct sensory modalities [103,104]. Although we are still far from the elucidation of mechanisms that cause schizophrenia, a role for the NMDA receptor has been suggested [105]. Experiments in rats clearly show that NMDA receptor antagonists can generate a dose-dependent selective impairment in multisensory information processing [106]. In another neuropsychiatric disorder, ADHD, adults with ADHD-like traits have reduced audio-visual integration window compared to those with low levels of ADHD-like traits. The authors suggested that malfunctions in perception of simultaneous stimuli could lead to the increased distractibility that characterizes ADHD [107].

Interestingly, the above neuropsychiatric diseases are all associated with difficulties in sensory processing and sociability. The mechanisms underlying this association are still unknown; however, there is strong evidence that dysfunctional multisensory integration might underlie aetiology and/or symptoms of a spectrum of neuropsychiatric disorders in humans.

## 6. Conclusion

**Figure 2.**
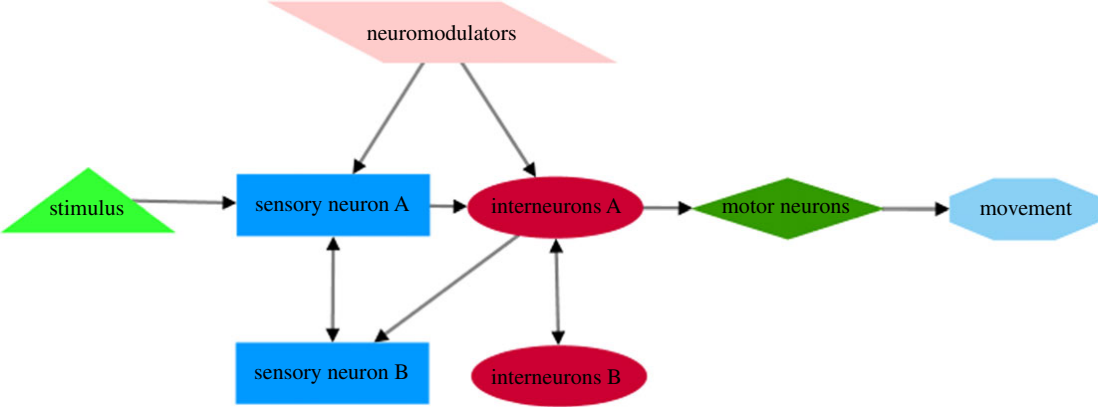
Schematic diagram of information flow during sensory integration in *C. elegans*. Interneurons integrate signals from multiple sensory neurons to produce appropriate motor output. Sensory neurons are indicated with blue rectangles, interneurons with red ellipses, motor neurons with green diamonds and motor output with light blue octagons. Light green triangle indicates sensory stimulus. Arrows denote flow of information through synapses or extrasynaptic interactions.

In. this review, we show that multisensory integration is a prominent mechanism through which *C. elegans* senses external stimuli and fine-tunes relative behavioural responses. In this complex network of interactions, a distinct category of nerve cells, the interneurons, have a distinguished role. Similarly to specific brain domains in mammals, interneurons are the decision-making centres where the flow of information from different modalities is converged and assessed. To initiate the most appropriate behavioural response, interneurons receive information regarding the organism's internal physiological state, through neuromodulators. These internal signals modulate activity of interneurons and, consequently, related responses according to the organism's immediate necessities. In this way, *C. elegans* takes threat–reward decisions according to its internal physiological conditions. Prior to the flow of information to interneurons, sensory neurons interact with each other and receive modulatory signals from the interior physiological systems. They can even form specific domains with interneurons, which resemble the ‘hub and spoke’ circuits, to ensure acute, automated and accurate responses (figure 2).

Research in *C. elegans* has the potential to elucidate basic rules governing multisensory integration in higher organisms, including humans. Recent evidence indicates a possible role for dysfunctional multisensory integration in the aetiology of certain neuropsychiatric diseases, such as ASDs. However, dysfunctional multisensory integration might underlie generally bad performance of the nervous system, including dizziness, balance problems and disorientation [108]. Hence, elucidation of mechanisms regulating multisensory integration will lead to a more precise and holistic view of how our nervous system functions and how it reconstructs the physical world in a coherent and unified depiction.

## Data accessibility

This article has no additional data.
